# Supplementary material for: Are carbon emissions trading and green financial instruments synergistic? -Comprehensive quantitative research based on content analysis
Source: PLoS One. 2024 Mar 7;19(3):e0298601. doi: 10.1371/journal.pone.0298601 (PMC10919601; doi:10.1371/journal.pone.0298601)
Supplement: S1 Data — (ZIP) [file pone.0298601.s001.zip › Data/地级市Z_F工作报告.zip!/╣π╢1⁄2/║╙╘┤╩╨/└┤╫╘╣1⁄2╓┌║┼í╛┬φ┐╦╩2╛▌═°í┐_2018.docx]

各位代表：

　　现在，我代表市人民政府向大会作政府工作报告，请予审议，并请市政协各位委员和其他列席人员提出意见。

一、2017年工作回顾

过去一年，我们紧密团结在以习近平同志为核心的党中央周围，深入贯彻落实党的十八大、十八届历次全会精神，认真学习贯彻党的十九大精神，以习近平新时代中国特色社会主义思想为指导，以习近平总书记对广东作出的重要指示批示精神为统领，在省委、省政府和市委坚强领导下，在市人大及其常委会和市政协监督支持下，坚持稳中求进工作总基调，坚持新发展理念，坚持以推进供给侧结构性改革为主线，乘势而上，攻坚克难，狠抓落实，推动全市经济社会平稳健康发展。

（一）经济保持平稳运行。坚持发展是第一要务，主动适应经济发展新常态，坚持问题导向，突出精准施策，推动经济平稳发展。预计实现地区生产总值996亿元、同比增长5.5%，其中一二三产业比重为9.9:44.6:45.5，第二产业、第三产业占比分别提高0.9和0.4个百分点；人均生产总值32280元、增长5.2%；规模以上工业增加值369亿元、增长4.3%；固定资产投资763亿元、增长17%；社会消费品零售总额585.8亿元、增长9%；进出口总额263.7亿元、增长1%；地方一般公共预算收入71.19亿元、增长5.7%；居民人均可支配收入17718元、增长10.2%；居民消费价格指数（CPI）涨幅为1.3%；民营经济增加值占GDP比重达65%。

（二）创新驱动促进产业转型升级。推动科技创新。实施高新技术企业倍增计划，高新技术企业达102家，增长54.6%，新增省创新型（含试点）企业5家。新增国家级、省级科技企业孵化器各1家。新组建省级工程技术研究中心38家、省级现代农业科技创新中心（基地）5家。135家规上工业企业建有研发机构、占比达22.7%，主营业务收入5亿元以上工业企业实现研发机构全覆盖。研发经费支出占GDP比重提高0.32个百分点，专利授权量增长60.8%，获省科学技术奖3项。促进工业转型。完成工业投资290.6亿元、增长15%。技改投资增长43.5%，组织89家企业实施机器换人、智能化改造和老旧设备更新。实现先进制造业增加值180亿元、增长4.3%，高新技术产业增加值118亿元、增长10.1%。扶持优势企业做大做强，新增规上工业企业64家，其中超亿元6家。实施质量强市战略，创省工业类名牌产品15个，制造业产品质量合格率达97%以上。推动现代服务业发展。现代服务业增加值占服务业比重达47%、提高3.8个百分点。建成省级服务业先进标准体系试点2个。积极创建“省级全域旅游示范市”，源城区、东源县成为省全域旅游示范区创建单位。接待入市游客3200.12万人次、增长12.1%，实现旅游总收入270.62亿元、增长13.8%。电商零售额50.6亿元、增长120%。推动企业上市，在资本市场挂牌企业96家。实施限额以上商贸企业倍增计划，新增限上企业100家。加快龙川铁路编组站物流园和市高新区综合物流园建设。推动现代生态农业发展。推进农业供给侧结构性改革，大力发展现代农业和特色农业，新增5个省级农业标准化示范区、20家市级以上农业龙头企业。灯塔盆地国家现代农业示范区核心区加快建设，灯塔盆地田园综合体入选国家首批试点项目。东源县入选第一批国家农业可持续发展试验区。超额完成省下达的粮食考评任务。加大耕地保护力度，加快建设高标准农田和垦造水田。

（三）“三大抓手”成效明显。交通基础设施建设持续发力。完成交通基础设施建设投资121亿元。汕昆高速（河源段，下同）建成通车、新增高速公路里程119.64公里，河惠莞高速全线动工，赣深客专、武深高速加快建设，龙梅龙客专、粤赣和惠河高速扩宽工程前期工作顺利推进。新增国道4条，超额完成国省道、县乡公路改造任务。完成河源通用机场预选场址规划，启动县区通用机场规划选址工作。东江航道恢复通航工作有序开展。产业园区扩能增效。完成扩园6.7平方公里，投入基础设施建设资金26亿元，完善一批市政道路、污水处理、供热管道等基础设施。133个优质项目落户，其中超亿元项目81个、超10亿元项目5个，新开工项目79个，新投产项目40个。深化园区合作共建，深圳投入共建园区资金19亿元，引进产业转移项目93个，中兴通讯（河源）基地启动搬迁。62家总投资额超过70亿元的模具企业集体签约落户深河国际模具城。中心城区扩容提质。启动新一轮城市总体规划修编，“三江六岸”、高铁新城等专项规划高标准开展。升级改造长安街、文明路、新风路、大同北路、小江桥下穿道路等一批市政道路。完成老城区39个背街小巷改造修缮项目。加快江东新区起步区建设，完成县道X168线临江至古竹公路项目建设。“西优”工程稳步推进。市区水源工程一期建成。新建、改建小公园43个，建成客家文化公园三期，启动东江湾公园二期建设，成功创建国家园林城市。市图书馆新馆工程获得中国建设工程鲁班奖。落实房地产调控政策，房地产市场运行平稳。

（四）深化改革增添活力。重点领域改革稳步实施。深化“放管服”改革，清理规范中介服务事项20项，取消权责事项99项，取消行政许可事项18项。推进“减证便民”，市本级取消证明事项112项。“一门式、一网式”政务服务创新平台全面投入使用。大力推动放权强区，授予江东新区、市高新区85项市级权限。启动“数字政府”建设。城市管理综合执法体制改革顺利推进。完成基本公共服务均等化综合改革试点工作，实施县（区）零基预算改革和财政资金项目库管理试点。完成普惠金融“村村通”试点工作，农信社改革工作顺利推进。全面推行“多证合一、一照一码”，强化事中事后监管，市场主体总量突破15万户，企业年报公示率排名全省第一。推进农村综合改革，农村土地确权登记颁证51.1万本，颁证率达99.1%、排名全省第三。东源、和平县成为省扶持村级集体经济发展试点县。完成3家国有企业公司制改革。“三去一降一补”扎实推进。完成去产能任务，取缔4家“地条钢”企业。化解非商品住房库存10.05万平方米。杠杆率控制在合理区间。出台降低实体经济企业成本工作方案，为企业减负超过36亿元。建立基础设施供给侧结构性改革补短板项目库。开放型经济要素加快集聚。开展投资项目审批改革试点，推行企业投资清单制、项目代办制，成功举办一系列重大招商推介活动，签约项目185个、总投资440亿元。出台支持出口退税融资服务促进外贸稳增长政策措施，实际利用外资增长9.5%。河源跨境电商监管中心启用，盐田国际河源内陆港投入运营，国际贸易“单一窗口”上线，柬埔寨商品河源体验馆开馆，“三互”大通关改革进一步推进，完成综合保税区选址工作。

（五）生态环境治理不断加强。坚决抓好中央环保督察反馈意见整改，全面落实“双控”、河长制等举措，环境质量持续好转。实施河源市“水十条”，新建污水处理厂10座、配套管网76.4公里，治理中小河流397.8公里，推进城市黑臭水体整治，12个国控、省控断面水质达标率91.7%，饮用水源达标率100%。47个新丰江水库生态环境保护项目完成36个。实施“五大治污”工程，开展大气环境综合整治“百日行动”，与深莞惠汕四市建立大气污染联防联控工作机制，超额完成省下达的“黄标车”三年淘汰任务，空气质量优良率达97.5%、综合排名全省第三。开展土壤污染状况详查工作。可完成省下达的节能降耗任务。实施新一轮绿化河源大行动，森林覆盖率74.6%。康禾温泉森林公园、东江湿地公园分别获得国家森林公园、国家湿地公园称号。龙川县建成省级林下经济扶贫示范县。东源县获评全国首批“绿水青山就是金山银山”实践创新基地。

（六）脱贫攻坚扎实推进。深入推进精准扶贫精准脱贫工作。落实各类扶贫项目5.9万个。打好扶贫政策“组合拳”，强化低保制度与扶贫政策有效衔接，推动教育、医疗、住房等扶贫政策落地到户。在全省率先出台扶贫项目资金快审快批、扶贫工作亮灯管理、驻村第一书记召回机制。两年累计投入精准扶贫资金19.39亿元。全面推行“两个全覆盖”，同步推进分散贫困人口脱贫工作。建档立卡的有劳动力贫困户人均可支配收入达7450元，44053人实现脱贫。深圳积极推动对口帮扶和精准扶贫工作，派出309名干部，投入对口帮扶资金37.98亿元，其中投入精准扶贫资金7.67亿元，结对帮扶河源214个贫困村。全面启动贫困村创建新农村示范村工作。完成255个贫困村整治规划编制工作。“三清理”“三拆除”“三整治”工作进展顺利，贫困村人居环境综合整治取得明显成效。完成省下达的5600户农村危房改造任务，修建硬底化村道512公里，新建水渠193公里，建成村卫生站152个。扎实推进面上新农村建设。紫金凤安、和平林寨、龙川丰稔省级新农村示范片建设顺利推进，累计完成投资3.53亿元。全市农村生活垃圾有效处理率达93.2%，村庄保洁覆盖率达100%，建成省级卫生村34个。建成宜居村庄、宜居城镇各7个，移民美丽家园示范点8个。原中央苏区682个行政村超高速无线局域网全面开通应用。紫金县获评首批“四好农村路”全国示范县。

（七）民生保障水平进一步提高。全年民生支出218.04亿元，占一般公共预算支出的77.1%。139项省、市民生实事顺利推进。教育“创现”扎实推进。源城区完成“省推进教育现代化先进区”申报。深入实施教育信息化工程和“强师工程”，推进“三通两平台”建设，教育质量持续提升。落实城乡义务教育寄宿制公办学校公用经费、家庭经济困难寄宿生生活费补助。新建、扩建8所中小学校，新增学位13420个；深圳中学河源实验学校基本完工。优化农村义务教育资源配置，撤并农村教学点69个；完成“全面改薄”工程269所学校的校舍建设任务，新改扩建校舍34万平方米。与广东技术师范学院共建河源校区，本科院校筹建工作顺利推进。卫生“创强”有序推进。深化医药卫生体制改革，取消公立医院药品加成，推进分级诊疗、医联体建设，启动4间城市公立医院改革。市人民医院二期建成使用、市精神卫生中心（市第二人民医院）正式运营，市妇幼保健院二期项目竣工，深河人民医院和东源、龙川、紫金县第二人民医院启动建设，19所乡镇卫生院实施标准化建设，基本完成334间村卫生站公建规范化建设。落实基本公共卫生服务项目，疾病预防控制和卫生应急处置能力进一步提升，计划生育管理服务得到加强，预计2017年人口自然增长率为8.5‰。文化体育服务水平逐步提升。大力推进市级公共文化设施社会化运营，公共文化服务体系不断完善。建成36个综合性文化服务中心示范点和10间特色文化室。实施文化惠民工程，深入开展丰富多彩的群众性文化活动。扎实推进精神文明建设，提升市民文明素质和城市文明程度，继续获得全国文明城市提名资格。开展粤赣古（驿）道勘测清理保护工作，文物保护利用、非物质文化遗产保护传承工作不断加强。全民健身运动广泛开展，成功举办河源越野挑战赛和第二届“河源万绿湖国际马拉松比赛”。社会保障不断完善。城镇新增就业4.3万人，城镇登记失业率控制在2.44%以内。深化机关事业单位养老和城镇职工医疗保险制度改革，推进全民参保登记计划，基本建立覆盖城乡居民的社会保险制度。实现深河异地就医定点互认。保障性安居工程稳步推进，新开工棚改安置住房1813套，基本建成棚改安置住房和公租房564套，新增分配公租房705套。完成村村通自来水工程65宗，惠及110.46万农村人口。全面完成村（社区）公共服务中心（站）建设。国防动员、双拥共建扎实推进，优抚安置政策全面落实。

（八）社会保持和谐稳定。构建风险分级管控和隐患排查治理双重预防机制，公共安全形势持续稳定。省矿山救援河源基地首期工程投入使用。推进智慧食药监项目和食用农产品快速检测项目建设，食品药品质量安全稳中向好。加强灾害防治能力建设，消除地质灾害隐患点50处，启动省地质灾害隐患点治理试点工作。在全省首创“智慧法援”平台，做好普法和一村（居）一法律顾问工作。基层治理不断强化，全面完成村（居）换届选举工作。实施全民禁毒工程。深入开展“飓风2017”专项行动，完成“中心+网格化+信息化”建设，推进立体化和信息化社会治安防控体系建设，刑事、治安案件发案数持续下降，“平安河源”基础进一步夯实，群众安全感不断增强。源城区获评“全国平安建设先进区”，源城公安分局获评全国优秀公安局。

（九）政府工作效能进一步提升。深入贯彻党的十八大、十八届历次全会精神，认真学习贯彻党的十九大精神，不断增强“四个意识”“四个自信”，坚决维护以习近平同志为核心的党中央权威，不折不扣落实省委、省政府和市委的决策部署。落实全面从严治党主体责任，深入推进“两学一做”学习教育常态化制度化，严格遵守中央八项规定精神，严厉整治“四风”，政府作风持续改善。加快政府职能转变，政务公开全面推行，政府决策机制日益完善，法治政府、阳光政府、廉洁政府和服务型政府建设不断推进。自觉接受市人大及其常委会的监督、市政协民主监督。强化督查督办，按照“分管领导抓、抓分管领导”的要求，建立健全对重点工作重点项目定人、定事、定时的“三定一督一通报”机制，发扬“钉钉子精神”，推动政府各项工作扎实落地。

与此同时，妇女儿童、民族宗教、外事侨务、对台、人民防空、史志档案、社会组织、殡葬管理、气象、地震、打私、供销等各项事业取得新进步。

各位代表！一年来的成绩来之不易。这是省委、省政府和市委正确领导的结果，是全市人民团结拼搏、共同奋斗的结果，是深圳、广州等兄弟市和海内外各界人士大力支持的结果。在此，我代表市人民政府，向全市广大干部群众，向驻河源部队、消防官兵和各民主党派、人民团体、社会各界人士致以崇高的敬意！向长期关心支持我市改革发展的港澳同胞、台湾同胞、海外侨胞表示衷心的感谢！

各位代表！我市经济社会发展虽然取得了一定的成绩，但对照党的十九大精神和中央、省的部署，对比高质量发展的要求，还存在一些问题和不足，主要是发展不平衡、不充分：一是产业基础仍然薄弱。传统低端产业占比大，高质量大项目、好项目不多，缺乏高新技术产业和产业集群。新业态新模式等新动能有待进一步培育。二是工业提速增效压力大。规上工业增加值增速低于年度目标5.7个百分点。工业投资增速低于同期房地产投资13.4个百分点。受去产能政策、环保整治和市场因素等影响，规上企业减产面达24.2%。三是县域经济发展滞后。各县区经济规模总体偏小，个别县生产总值仍未破百亿。主要经济指标绝对值大小不一、增速差异明显，工业增加值增速最快和最慢的园区之间相差12倍。四是“三农”欠账多。村居环境脏乱差现象普遍，农村生产生活配套有待完善。农业现代化水平不高，新型农业经营主体数量较少，有影响力的农业品牌不多。农民收入水平明显偏低，贫困人口稳定脱贫机制仍不健全。五是城市配套不够完善。城区部分学校大班额现象依然存在，优质学位依然紧张。优质医疗资源短缺，医疗服务水平有待提高。道路承载能力不足、交通秩序不优，行车难、停车难问题突出。六是部分领域风险因素不容忽视。安全生产工作存在不少薄弱环节，高危行业和易发事故的领域多、潜在风险大。食品药品制假隐蔽化、售假网络化，事故防控难度加大。七是环境保护力度有待加大。污水处理设施不足，垃圾处理能力不强，城市建成区黑臭水体整治不彻底。部分矿山环境治理和生态修复滞后。八是政府职能转变和作风建设还有差距。政务服务水平不高，投资营商环境亟待改善。一些干部干事创业精气神不足，攻坚克难的创新办法不多。我们要直面这些问题，采取更有力的措施加以解决。

二、2018年重点工作安排

2018年是贯彻党的十九大精神的开局之年，是改革开放40周年，是决胜全面建成小康社会、实施“十三五”规划承上启下的关键一年，是河源建市30周年、步入乘风远航快速发展的一年，做好今年政府工作意义重大。今年政府工作的指导思想是：全面贯彻党的十九大和中央经济工作会议精神，以习近平新时代中国特色社会主义思想为指导，认真落实习近平总书记重要指示批示要求及省委十二届二次、三次全会和市委七届三次、四次全会决策部署，坚持稳中求进工作总基调，坚持新发展理念，紧扣我国社会主要矛盾变化，贯彻高质量发展的要求，统筹推进“五位一体”总体布局和协调推进“四个全面”战略布局，坚持以供给侧结构性改革为主线，统筹推进稳增长、促改革、调结构、惠民生、防风险各项工作，全面提升创新、协调、绿色、开放、共享发展水平，推动质量变革、效率变革、动力变革，在防范化解重大风险、精准脱贫、污染防治上取得扎实进展，在交通设施建设、园区扩能增效、城市扩容提质上取得更大成效，协调推进生态河源与现代河源、都市经济与县域经济、城镇建设与乡村振兴，促进经济社会持续健康发展，推进建设粤港澳大湾区东北部核心城市，为决胜小康、率先振兴和同步现代化打下更坚实基础。

今年经济社会发展的主要预期目标是：地区生产总值增长7%左右，规模以上工业增加值增长7%，固定资产投资增长15%，进出口总额增长3%，社会消费品零售总额增长9%，地方一般公共预算收入增长8%，居民消费价格涨幅控制在3%以内，城镇登记失业率控制在3%以内，城乡居民收入增长与经济增长基本同步，主要污染物总量减排和大气、水环境质量完成省下达的目标任务，供给侧结构性改革取得实质性进展，金融财政风险有效防控。

为实现今年各项目标任务，重点抓好九方面工作：

（一）深化供给侧结构性改革，建设现代化经济体系。

坚持从供给侧发力，以实体经济振兴为着力点，全力推动经济向高质量发展阶段迈进。

大力推动园区扩能增效。科学调整“一区六园”功能布局，加快形成以市高新区为龙头、中心城区“四园”联动发展、各县园区错位发展的格局。完善深河两市产业合作共建方案，健全园区共建共享机制，鼓励各园区和深圳各区结对帮扶，因地制宜引进项目，实现新落户、新开工、新投产项目各50个以上。用好省产业共建财政政策，加快园区扩园，补齐园区配套短板。深入推进“三去一降一补”，落实好国家“降税减证提标”三项措施、省支持实体经济发展的10项措施，促进企业综合成本合理下降。继续实施规上企业倍增计划，新增规上企业70家以上。实施新一轮技改三年行动计划，推动80家以上企业开展技改，完成技改投资84亿元以上。培育壮大新一代电子信息、高端装备制造、新材料、新能源、生命健康等新兴产业。深入实施市、县领导挂扶企业机制，强化产业园区目标管理，强化在建项目跟踪服务，推动项目加快履约开工。加快中兴通讯（河源）基地搬迁投产和深河国际模具城建设。

大力发展现代服务业。加快发展现代物流、现代金融、电子商务、云计算服务、信息咨询、专业市场和会展经济等生产性服务业；促进商贸服务、文化产业、健康服务等生活性服务业提速发展。大力实施限上商贸企业倍增计划，新增限上企业110家；培育一批跨境电商和农村电商示范县、示范镇村。全力做大生态旅游业，着力谋划全市旅游“一带一圈”发展格局。加快创建省级全域旅游示范市、县，推动“旅游＋”跨界融合，深度挖掘和提升龙川五兴龙苏维埃政府旧址、紫金苏区等红色文化旅游，高标准打造旅游特色街区和一批乡村旅游示范单位，推动特色民宿产业发展，加快筹建东江百里旅游长廊。提升万绿生态旅游产业园，推动万绿湖创建5A景区和国家生态旅游示范区、巴伐利亚庄园创建国家级旅游度假区。提高旅游公共服务能力，狠抓“厕所革命”，加快智慧旅游平台建设，推动一批游客集散中心建设。加大在粤港澳大湾区城市的推介力度，举办好第十三届客家文化旅游节，擦亮河源旅游品牌。

加快发展民营经济。着力营造宽松平等的准入环境，鼓励和引导民间资本进入基础设施和市政公用事业等领域，释放民企发展活力。落实中小企业信贷风险补偿金、联合科技信贷风险准备金、政策性融资担保和小额贷款保证保险资金等支持政策，解决中小微企业发展融资难、融资贵问题。落实财政资金支持举措，完善上市后备企业资源库，鼓励企业利用资本市场做大做强。加强纳税信用评价结果的增值应用，为符合条件的企业提供信用贷款。鼓励外出乡贤回乡创业投资，扩大民企投资规模。提升民营企业创新能力，培育一批民营科技企业。切实保护民营企业合法权益，激发和保护企业家精神，积极构建“亲”“清”新型政商关系。大力培育大型骨干企业，健全中小微企业服务体系，支持“个转企”“小升规”，扶持民营企业做大做强。

加快推进基础设施建设。深化基础设施供给侧结构性改革，完善项目库建设，争取更多项目纳入省项目库。大力推进127项年度计划投资313.35亿元的省、市重点项目建设。构建现代综合交通运输体系，努力建设粤港澳大湾区东北部交通枢纽。加快建设河惠莞、武深等高速，推进粤赣高速埔前至热水段扩建工程，推动河紫高速等规划实施，积极谋划河惠深、河增、连梅韶、龙寻高速；加快赣深客专建设，力争龙梅龙客专开工建设，谋划建设广河客专、龙（川）韶（关）铁路、龙（川）汕（尾）铁路等重大铁路项目；推进支线机场和通用机场规划建设，推动东江航道河源段扩能升级和恢复通航。推进国、省道升级改造，加快国道G236线紫金县城段、国道G238线和平县城段、省道S243线东源县地运至蓝口段等建设，推动国道G205线热水至埔前段、国道G205线龙川县城段、省道S253线忠信至青州段、省道S253线顺天至灯塔段等开工建设；加快规划建设市区连接各县城的高快速通道，推进高速公路、高铁出入口与产业园区道路和城市主干道的连接通道建设。抓好“四好农村路”建设，实现95%的行政村“村村通班车”。推进老旧小区光纤入户改造，启动市区43个公共免费无线局域网和640个无线接入点建设。推进中小河流治理、村村通自来水、高效节水灌溉项目建设，完善江河水库防洪体系。加快市高新区智慧园区项目建设，推进河源电厂二期、农村电网、天然气管网和电动汽车充电设施建设。

（二）大力实施乡村振兴战略，坚决打赢脱贫攻坚战。

坚持农业农村优先发展，坚持农民主体地位，坚持乡村全面振兴，推动农业全面升级、农村全面进步、农民全面发展。坚持把打赢精准脱贫攻坚战作为头等大事，提高脱贫的质量和成效。

深入推进农业供给侧结构性改革。坚持质量兴农、绿色兴农，实施质量兴农战略，不断提高农业创新力、竞争力和全要素生产率。加快灯塔盆地国家现代农业示范区核心区建设，推进灯塔盆地田园综合体国家试点建设工作，夯实农业生产能力基础。支持农业龙头企业和农民专业合作社开展农产品加工，新增市级以上农业龙头企业15家，农民专业合作社350家，加快推进农业由增产导向转向提质导向。加大良种良法推广应用，提高种粮大户的积极性，确保粮食生产稳产、增产。加快传统农业改造步伐，培养造就一支懂农业、爱农村、爱农民的“三农”工作队伍。多渠道推进涉农资金整合，采取政府和社会资本合作、以奖代补和贴息等形式，吸引更多资本投向农业。支持返乡农民工、高校毕业生等创办领办新型农业经营主体，引导发展休闲农业和乡村旅游等农村新产业新业态，培育引进一批集聚效应强的多层次涉农电商平台，着力构建农村一二三产业融合发展体系。落实耕地占补平衡，加快高标准农田建设。

深化农村重点领域改革。巩固和完善农村基本经营制度，完善承包地“三权”分置制度，继续推进农村土地承包经营权确权登记颁证工作，明确农民对土地承包权、宅基地使用权和集体收益分配权的权属。建立符合市场经济要求的集体经济运行机制，确保集体资产保值增值，确保农民受益。抓好农村集体产权制度改革试点工作，健全村组两级集体经济组织运行机制，鼓励以土地股份合作、土地托管等方式，拓宽放活土地经营权。拓展农村产权流转管理服务平台功能，规范土地经营权流转交易程序。稳步扩大农村集体资产股份权能改革试点范围，提高农村各类资源要素的配置和利用效率，多途径发展壮大集体经济。鼓励多渠道筹集资金，支持扩大农产品价格指数保险试点工作。持续推进农业保险扩面、增品、提标，采取以奖代补方式支持开展特色农产品保险，扩大主要农产品政策性农业保险覆盖面。

确保建档贫困人口如期脱贫。进一步提升产业扶贫，引导贫困村发展主导产业，带动贫困户建立稳定增收渠道。因地制宜推动贫困劳动力转移就业，增加劳务收入。积极盘活贫困村土地、水利、房产、森林等资源，增加贫困户资产性收入。完善工作机制，推动扶贫政策全面落地到户。把扶贫和扶志、扶智相结合，激发贫困人口内生脱贫动力，巩固扶贫成果，提高脱贫质量。扎实推进中央和省属水库移民后期扶持政策，实现在册移民贫困户如期脱贫。开展企业帮扶农村发展行动。开展扶贫领域腐败和作风问题专项治理。强化各级党政主抓扶贫责任，加强驻村工作队管理，实行最严格扶贫考核，确保脱贫取得实效。

全面推进美丽宜居乡村建设。大力实施村容村貌提升、农村环境综合整治、农村基础设施建设、富民增收、管理创新五大工程，255个贫困村达到省定环境整治标准。以整治为主、整治与建设相结合，集中整治农村污水、垃圾、旧房等问题，提升村道通行水平，基本完成集中供水、雨污分流、垃圾处理等基础设施建设，消除“脏乱差”，健全保洁管护长效机制，实现村庄整洁有序宜居。把客家文化深度融入乡村建设，打造具有客家文化风情的美丽乡村。发挥农民主体作用，鼓励村民共谋共建共管共享新农村建设。鼓励有条件贫困村率先建成新农村示范村。鼓励和扶持以省定贫困村创建新农村示范村为基础，连片规划建设新农村示范片。完善紫金、和平县两个省级新农村示范片建设，加快龙川县省级新农村示范片建设，推进东源县省级新农村示范片建设。加快10个移民村美丽家园建设。创建一批省市级卫生村。

（三）实施创新驱动战略，加快新旧动能转换。

坚持创新是引领发展的第一动力，培育壮大新动能，推动经济从要素驱动、投资规模驱动发展为主向创新驱动发展为主转变。

培育企业创新主体。落实高新技术企业“树标提质”行动计划，支持骨干企业创建省级创新型试点企业和高新技术企业百强企业。落实好省推进高新技术企业培育等八大举措，重点实施高新技术企业倍增计划，力争高新技术企业增加到140家。实施科技型中小微企业成长计划，推动数量和质量双提升。完善科技企业孵化育成体系建设，大力建设省级以上科技企业孵化器。

推进科技创新平台建设。大力培育高端研发机构，争取建设国家级工程技术研究中心、广东省企业重点实验室、院士博士工作站。加强新型研发机构建设，推进河源市省科院研究院和河源广工大协同创新研究院建设。启动广东技术师范学院协同创新研究院建设。新组建一批省、市级工程技术研究中心和现代农业科技创新中心，实现规上企业建有研发机构比例达到25%以上，推进专业镇产业协同创新中心建设。

加强科技创新合作。加强区域协同创新，主动融入广深科技创新走廊，积极对接广深科技体系和科技市场，建立科技成果转移转化服务平台，组织开展科技成果对接活动，推动广深等珠三角地区科技成果在我市转化落地。深化军民科技融合发展，推进砺剑防务集团等军民融合项目落地，加快军民两用技术成果转化。借助深莞惠经济圈（3+2）平台，积极参与五市跨行政区域科技创新孵化育成体系建设，主动承接深莞惠创新资源溢出，引导新型研发机构、高等院校和科研院所入驻。引导企业走出去，组织企业参加中国高校科技成果交易会和中国创新创业成果交易会等，寻求科技创新合作。

营造良好创新环境。落实研发投入后补助、研发费用加计扣除、科技创新券等扶持政策，激励企业加大研发投入。促进科技金融融合发展。继续做好“珠江人才”“扬帆计划”申报工作，引进适合我市产业发展的创新创业团队和科技领军人才。强化知识产权创造、保护、运用，促进发明创造增量提质，加大专利执法维权，搭建专利质押融资平台。大力推动“大众创业、万众创新”，营造创新创业良好氛围。

深入推进质量强市建设。树立质量标杆，开展第二届河源市政府质量奖评审，继续开展“广东省质量强市（县、区）示范城市”和“广东省知名品牌示范区”创建活动。发挥质量创新动力，着力在支柱产业、传统优势产业、战略性新兴产业等领域培育一批名牌产品。重点推进热泵空调产品、塑料模具产品等省级质量监督检验站建设。

（四）融入粤港澳大湾区建设，构建开放型经济新体制。

把对外开放的重心和着力点转向粤港澳大湾区，促进高端要素集聚，加快发展更高层次的开放型经济。

促进开放型经济要素集聚。主动参与粤港澳大湾区开放型经济分工，积极承接粤港澳大湾区供应链分拨功能，加快推进物流中心、跨境电商监管中心、综合保税区建设。深入推进口岸“三互”大通关，提高国际贸易“单一窗口”覆盖率，提升贸易便利化水平。加强与广深机场、港口等大型物流节点对接，大力发展多式联运，做好公路、铁路、水路文章。进一步调整优化外贸结构，努力开拓国内外市场，促进外贸转型升级，培育市场采购、外贸综合服务和服务外包等新业态，推动现代化元素集聚。

大力优化营商环境。加快与国际通行规则衔接，对标粤港澳大湾区，打造国际化、市场化、法治化、便利化营商环境。强化政策支持，落实省“外资十条”和出口退税、出口信保、便利通关等政策，探索开展政策法规、产业准入、政务服务、技术标准等对接工程，以政策“洼地”支撑投资“高地”。强化要素保障和服务支撑，优化信息咨询、资质认证等服务功能，向投资者提供项目代办、投融资等全链条式服务，完善外商投资管理体制，以政府服务的“加法”换取市场活力的“乘法”。着力把市高新区和江东新区打造成优化营商环境的“示范区”。

完善招商引资体系。健全领导牵头招商、市县两级联动招商、职能部门共同招商、社会力量参与招商的综合招商机制。创新招商方式，引资、引智、引技并举，大力开展专业招商、产业招商、中介招商、产学研招商等立体招商，重点到9+2粤港澳大湾区各城市开展路演。发挥好招商顾问委员会和咨询委员会作用，建设高素质招商队伍，提高专业招商水平。围绕粤港澳大湾区的产业延伸和功能拓展，结合我市产业发展战略定位、功能布局和企业发展的需求，进一步开展主导产业研究，完善招商项目信息库，建立招商引资对接平台。

（五）实施都市经济带动战略，促进区域协调发展。

统筹城乡发展与区域发展，大力发展都市经济和县域经济，建立健全更加有效的区域协调发展新机制。

加强城乡规划管控。加强规划编制工作，继续推进规划期至2035年的城市总体规划编制工作，加强部门协调和城乡统筹，实现城市各类空间规划“一张图”管理。完成海绵城市、综合管廊、“三江六岸”生态景观城市功能廊道规划设计和高铁新城相关规划成果的编制工作。强化城建工作体系建设，优化体制机制，明确管理边界和职责分工，做好城市运营。完善城市规划建设管理制度，提高城乡规划建设管理水平。强化城乡规划管控督查，完成城乡规划建设管理队伍和城乡规划编制全覆盖工作，继续推进市派县城乡规划督察员工作。

加快中心城区提质扩容。优化城市功能布局，以“三江六岸”生态景观城市功能廊道为抓手，统筹老城区、城市北区、东源县城、江东新区、市高新区、城市西部六大片区发展。加快江东新区建设，推动新区纳入深圳河源产业共建范围，推进城市起步区、产业园区建设，打造中央商务区，逐步形成城市发展的核心。推动老城区提质，实施“百街千巷”改造提升工程，启动老城“四宝”文化绿道建设。加快实施“西优”工程，继续推进宝源新江片区基础设施项目和市区水源工程、供水工程建设。加快市政基础设施建设，实施新一路建设以及建设大道下穿中山大道、文昌路工程，推动中山大道、庄田桥、宝源桥等道路、桥梁改造。提升城市园林绿化水平，启动客家文化公园四期建设，完成东江湾公园二期、黄子洞公园、客家乡土植物园建设。

加快县域经济发展。协同推进工业化、新型城镇化和农业农村现代化，强化县域经济多点支撑，统筹推进城乡一体化。实施主体功能区规划，坚持分类指导，引导各县区因地制宜优化产业布局，培育一批辐射带动作用强的特色产业和产业集群，推动各县区形成1-2个特色支柱产业。促进各县区主动接受中心城区的辐射带动，形成更加科学紧密的经济协作和产业协同关系，主动对接粤港澳大湾区国家战略，大力推进与珠三角产业共建，加快形成环市区电子信息、装备制造等高端产业集聚地。坚持产城融合、产业兴城、城园互动，推动工业、服务业与城镇建设同步发展，增强城镇产业承载与服务能力，促进农村富余劳动力向城镇和非农产业转移，进一步促进产城人融合。扎实推进现代农业产业园建设，着力培育打造特色农产品品牌，扶持发展“一村一品”“一镇一业”，推动各县区培育1-2个专业镇。加快建设“特色小镇、美丽乡村”，推动各县区培育1-2个特色镇村。

（六）坚持绿色发展，建设美丽河源。

坚持“尊重自然、顺应自然、保护自然”，进一步巩固提升生态优势，打造生态河源。

加强生态保护。加强重点生态系统保护，提升生态系统质量和稳定性。完成生态保护红线、永久基本农田、城镇开发边界三条控制线划定工作。统筹推进山水林田湖草系统治理。强化湿地保护。全面推进省地质灾害隐患点治理试点工作。继续推进新一轮绿化河源大行动，加快林业重点生态工程建设，强化生态公益林和自然保护区管理，完善天然林保护制度，不断提升森林生态功能。稳步推进乡村绿化美化工程和森林小镇建设，建设宜居生态家园。积极推动纯松林和水源地桉树林改造，改善重点生态敏感区域生态环境。严格保护和科学利用林地资源，服务经济社会发展。强化森林防火工作，推动林火远程视频监控全覆盖。积极创建全国水生态文明城市。开展创建节约型机关、绿色家庭、绿色学校、绿色社区和绿色出行等活动。

加强环境治理。继续推进落实中央环保督察反馈意见整改。坚决打好大气、水、土壤污染防治“三大战役”。强化大气污染防治，大力推进“五大治污”工程，全面开展挥发性有机物排放治理，继续推广使用新能源汽车。持续推进水污染防治行动，继续开展南粤水更清行动，实施新丰江水库水质保护三年行动计划，确保新丰江水库水质保持国家地表水Ⅰ类；综合整治东江新丰江市区段水环境，完成东埔河黑臭水体整治任务，继续推进三王坝河、金竹沥、鳄湖、高埔小河、黄沙河等河流整治工作；加强龙川县鹤市河水环境整治，确保莱口断面水质稳定达标；以PPP模式整县推进镇村污水处理项目建设，确保2019年实现全市乡镇污水处理设施全覆盖、80%的农村生活污水得到有效处理。深入实施河源市“土十条”，开展土壤污染源详查，摸清污染状况底数，加快建设农用地土壤环境质量基础数据库，强化农业面源污染防治，切实管控土壤环境风险。持续削减污染物排放量，完成污染减排目标任务。启动第二次全国污染源普查工作。

加强生态文明制度建设。继续全面落实河长制，建立健全“一河（湖）一策”治理管护长效机制，实现“河畅、水清、堤固、岸绿、景美”目标。主动争取省制定框架办法，积极推进东江流域生态补偿机制建设。实行环境与发展决策咨询制度，推动建立跨省、市水质保护联席会议制度，完善环保综合决策机制。实施控制污染物排放许可，建立资源环境承载能力监测预警、环保准入负面清单和污染产业环保退出、环境污染责任保险等机制，发挥环境保护税的调节作用，构建齐抓共管的生态环境治理体系。

（七）加大改革攻坚力度，增强率先振兴动力。

坚持全面深化改革，坚决破除一切不合时宜的思想观念和体制机制弊端，为率先振兴发展提供强劲动力。

扎实推进“放管服”改革。以高新区和江东新区为试点，深入推进全市商事制度改革；推进“证照分离”改革，简化市场准入手续；全面落实市场准入负面清单制度和公平竞争审查制度。加快推进政务服务管理体制改革，全面推行“三集中三到位”行政审批管理模式，实行行政审批标准化；清理调整行政审批服务事项，减少审批环节，缩短审批时限，建立健全行政审批和公共服务事项目录动态管理运行机制。启动市民服务中心大楼建设，推进政务信息化建设体制改革，构建“管运分离”的“数字政府”，建设“互联网+政务服务”体系。深化城市管理综合执法体制改革，推进城市治理网格化，全面提高执法效能。深化事业单位分类改革，分批分步推进行政职能和经营服务类事业单位改革。

加快推进国资国企改革。完善各类国有资产管理体制，有序有效推进混合所有制改革。健全国企现代企业制度，通过资源整合、布局调整、产权优化、监管强化，推动国有企业加快发展。按“一企一策”原则，妥善处理国有企业资产保全、资产盘活和债权债务等问题，防止国有资产流失。

深入开展财税金融体制机制改革。加快现代财政制度改革，坚持“全面规范透明、标准科学、约束有力”原则，继续全面深化零基预算、财政资金项目库管理等预算制度改革，全面实施绩效管理，提高财政资金的使用效益。深化税收征管体制改革，提高管理服务水平。深化金融体制改革，增强金融服务实体经济能力。完成农信联社改制工作。引导和鼓励银行业金融机构创新金融服务，加大对先进制造业、战略性新兴产业、现代服务业以及产业转型升级、企业技术改造等领域的资金支持。全面推广农村普惠金融试点工作，增强农村金融服务能力。

切实做好风险防范化解工作。加强政府债务管控，强化政府性债务限额管理、预算管理和分类管理，规范政府购买服务行为。加强金融监管，确保金融机构杠杆率控制在合理区间。建立金融风险防控机制，着重做好防范处置非法集资、互联网金融风险专项整治等工作。坚持“房子是用来住的，不是用来炒的”定位，加快建立多主体供应、多渠道保障、购租并举的住房制度，合理控制商品房供应规模，引导投资行为，强化市场监管，保持房地产市场稳定。

（八）坚持以人民为中心，打造人民幸福“心城”。

以造福人民为最大政绩，想群众之所想，急群众之所急，让人民生活更加幸福美满。

优先发展教育事业。加大教育“创现”力度，实现广东省推进教育现代化先进县（区）全覆盖。引进基础教育品牌学校到河源办学，加快华南师范大学附属河源学校建设，推动深圳中学河源实验学校如期开学。增加市区和县城义务教育学位供给。开展农村义务教育寄宿制学校建设试点工作，稳妥推进乡村小规模学校（含教学点）撤并。发展素质教育，落实立德树人根本任务。加快推进教育信息化，推动优质教育资源共享。继续实施“强师工程”，推进农村小学教师培养机制改革，探索“县管校聘”和“校长职级制”改革，提升教师队伍素质。继续实施学前教育三年行动计划，促进学前教育公益普惠发展。实施第二期振兴初中行动计划。深化产教融合、校企合作，增强职业教育服务地方经济发展的能力。加快广东技术师范学院河源校区建设。落实《民办教育促进法》，推进民办学校分类管理改革，规范民办教育，提高民办教育水平。

继续推动卫生“创强”。实施健康河源战略，创建卫生强市。加快推进深河人民医院、东源县人民医院新院等建设。加强全科医生、学科带头人等重点人才培养和储备，强化专业培训，推进临床重点专科建设，创建省级重点专科，提升卫生服务能力。深入推进医药卫生体制综合改革和医联体建设，落实基本公共卫生服务项目。按照“非禁即入”原则，促进社会办医。建立健全公共卫生安全体系，增强重大疾病防控能力，提升突发事件卫生应急能力。加强“全面两孩”政策实施的配套服务。做好国家卫生城市复审工作。

加强公共文化建设。探索政府购买公共文化服务途径，实行市“四馆”社会化运营管理，推进文化馆、图书馆总分馆制建设和公共文化服务体系示范区（项目）建设。继续实施文化场馆（站）等级提升工程，实现行政村（社区）综合性文化服务中心全覆盖。深入实施文化惠民工程，提高文化场馆免费开放效能，积极开展送戏下乡、送书下乡、农村公益电影放映活动，继续推进数字档案馆（室）建设。积极推动文化产品开发和文化产业发展，传承弘扬革命精神，保护修缮一批红色革命遗址。加强文物保护利用和非物质文化遗产保护传承，加大粤赣古（驿）道开发力度，开展河源恐龙博物馆展陈提升工程建设，完善重点文物“四有”保护工程。抓好版权保护开发，推进正版软件使用。启动新一轮创建全国文明城市工作，力争2020年成功创建。

加强社会保障体系建设。坚持就业优先战略，突出抓好高校毕业生、就业困难人员、失业人员等重点群体就业，促进女性平等就业，鼓励创业带动就业，力争新增城镇就业44400人，安置下岗失业人员再就业7000人、就业困难人员再就业1000人，促进创业1800人，带动就业5000人。推进工业园区人力资源服务产业园建设。全面实施全民参保计划，加强社保扩面征缴，稳步提高社保待遇水平。全面落实机关事业单位养老保险制度改革工作。健全全民医保制度，完善统一的城乡居民基本医疗保险制度和大病保险制度，完善不同级别医疗机构医保差异化支付政策，扩大医保异地就医即时结算范围。完善最低工资标准调整和工资支付保障制度。加强社会救助体系建设，全面开展重特大疾病医疗救助。完善最低生活保障制度。提高优待抚恤标准。加强残疾康复服务，推进残疾人事业发展。扎实抓好住房保障工作，基本建成各类棚改安置住房224套，完成省下达的目标任务。

营造和谐社会环境。牢固树立总体国家安全观，深入推进平安河源建设，提升群众安全感。完善矛盾纠纷多元化解机制，有效排查不稳定因素。夯实基层治理基础，加强公安派出所建设，继续推进“一村（居）一辅警”；突出科技支撑、信息主导，发挥“中心+网格化+信息化”作用，全面推进“五星”平安村（社区）建设。健全社会治安防控体系，推进“雪亮工程”建设，盯紧社会治安突出问题，深化严打整治行动，全面推进全民禁毒工程。加强重点人群、重点行业场所、危险物品管理。健全公共安全体系，落实安全生产责任制，加强事故隐患源头治理，提升防灾减灾救灾能力。落实食品药品安全责任制，加快建设市食品药品检验技术中心，启动源城区创建广东省食品安全示范县工作，保障食品药品安全。加大全民普法力度，推进公共法律服务体系建设。加强社会信用体系建设。全面开展第十一届“全国双拥模范城”创建活动。

继续做好妇女儿童、民族宗教、外事侨务、对台、人民防空、史志档案、社会组织、殡葬管理、老龄、气象、地震、打私、供销等工作。

扎实办好十件民生实事：投入各类资金72.46亿元，其中市级以上财政投入37.57亿元，办好十件民生实事。一是提高山区边远地区学校教师生活补助。二是改善城乡居民生产生活条件。三是全面提升交通安全水平。四是推进新丰江、东江水质保护。五是完善教育基础设施建设。六是加强基层医疗卫生服务体系建设。七是实施农村适龄妇女“两癌”免费检查。八是实施市区“百街千巷”改造提升项目。九是实施“旅游厕所革命”。十是推进红色文化遗址修复工作。（详见附件）

（九）加强政府自身建设，努力建设人民满意政府。

坚持和加强党的全面领导，加快政府职能转变，提高政府执行力。

落实全面从严治党要求。以习近平新时代中国特色社会主义思想为指导，认真学习贯彻党的十九大精神和新《党章》，牢固树立“四个意识”，坚决维护习近平总书记的核心地位，始终在政治立场、政治方向、政治原则、政治道路上同以习近平同志为核心的党中央保持高度一致。认真贯彻《中国共产党党组工作条例（试行）》，全面落实政府党组主体责任。认真贯彻中央八项规定精神，严防“四风”反弹。严格执行《中国共产党党内监督条例》，不断完善政府权力运行监督机制，全面加强行政监察和审计监督。以零容忍态度惩治腐败。

确保权力规范阳光运行。认真执行新形势下党内政治生活若干准则，健全落实重大行政决策机制，建立健全“三重一大”集体决策制度，发挥政府法律顾问参谋作用，推进行政决策科学化、民主化、法治化。落实规范性文件管理新要求。全面落实行政执法责任制，严格规范公正文明执法。落实行政复议开庭审理制度，全面推行行政机关负责人出庭应诉工作，发挥行政复议意见书和建议书作用，强化行政复议的延伸监督。落实重大事项报告制度。自觉接受市人大及其常委会的监督，广泛听取各民主党派、工商联、无党派人士和各人民团体的意见，将行政权力运行关在监督的“栅栏”内。依法深入推进政务公开。

驰而不息改进工作作风。把工作标准调到最高、把精神状态调到最佳、把办法措施调到最优，撸起袖子加油干，有力有效推动工作。坚持狠抓作风建设不松懈，扎实推进“两学一做”学习教育常态化制度化，深入开展“不忘初心、牢记使命”主题教育和“大学习、深调研、真落实”活动，大力推行“三定一督一通报”，大力倡导“钉钉子精神”，精准破解发展中的痛点难点。鼓励符合中央精神、改革方向以及法律法规的创新性行为，激发干事创业热情，推动工作再上新台阶。

各位代表!团结凝聚力量，奋斗成就伟业。让我们更加紧密地团结在以习近平同志为核心的党中央周围，在市委的领导下，不忘初心、牢记使命，同心同德、真抓实干，奋力完成今年经济社会发展各项目标任务，为决胜小康、率先振兴、同步现代化而努力奋斗!
